# Comparative effectiveness of initiating insulin versus metformin therapy for gestational diabetes mellitus in China: a retrospective cohort study

**DOI:** 10.1186/s12884-026-09752-4

**Published:** 2026-07-31

**Authors:** Linfeng Jiang, Nan Peng, Mengyao Xue, Ruolan Wei, Guoxian Lu, Yuqing Fan, Shuo Zhang, Beini Lyu, Dongning Yao

**Affiliations:** 1 Department of Pharmaceutical Regulatory Science and Pharmacoeconomics, School of Pharmacy, Nanjing Medical University, Nanjing, Jiangsu 211166 China; 2 School of Pharmaceutical Science and Technology, Tianjin University, Tianjin, China; 3 Institute for Global Health and Development, Peking University, Beijing, 100871 China; 4 Department of Pharmacy, The Second People’s Hospital of Changzhou, The Third Affiliated Hospital of Nanjing Medical University, Changzhou, Jiangsu 213004 China

**Keywords:** Gestational diabetes mellitus, Insulin, metformin, Retrospective cohort study, Glycemic control, Pregnancy outcomes

## Abstract

**Background:**

While insulin is widely recommended as the first-line pharmacological therapy for gestational diabetes mellitus (GDM), the use of metformin is increasing in routine clinical practice. However, real-world evidence comparing their effectiveness and safety remains limited. This study aimed to evaluate glycemic control and adverse pregnancy outcomes between the insulin and the metformin initiation group in women with GDM in China.

**Methods:**

We conducted a retrospective cohort study using electronic medical records from a provincial healthcare database in Eastern China. Women diagnosed with GDM between 2018 and 2025 were included. Patients were divided into the insulin initiation group and the metformin initiation group based on their first prescription. Propensity score overlap weighting (OW) was used to balance baseline characteristics. Weighted linear and logistic regression models were used to assess glycemic outcomes, including the glycated hemoglobin (HbA1c) level at the end of pregnancy, the change in HbA1c, and target achievement (HbA1c < 6.0%). Adverse pregnancy outcomes were evaluated using weighted Cox proportional hazards models with gestational age as the time scale.

**Results:**

Of the 7,397 women included, 6,922 (93.6%) initiated insulin and 475 (6.4%) initiated metformin therapy. After OW adjustment, no significant differences were found between the two groups in end-of-pregnancy HbA1c levels, HbA1c changes, or glycemic control rates. Compared with insulin initiation, metformin initiation was associated with significantly lower risks of cesarean delivery (HR: 0.77, 95% CI: 0.62–0.95; *P* = 0.017), premature rupture of membranes (HR: 0.66, 95% CI: 0.48–0.93; *P* = 0.016), and macrosomia (HR: 0.61, 95% CI: 0.40–0.93; *P* = 0.021). Risks for other adverse outcomes, including preterm birth and preeclampsia, were comparable. Sensitivity analyses, including per-protocol analysis, confirmed the robustness of these findings.

**Conclusions:**

In this real-world Chinese cohort, the metformin initiation group achieved glycemic control similar to the insulin initiation group. In addition, the metformin initiation group had lower risks of cesarean delivery, premature rupture of membranes, and macrosomia. These findings suggest that metformin may be a viable and effective initial treatment option for selected women with GDM.

**Supplementary Information:**

The online version contains supplementary material available at https://doi.org/10.1186/s12884-026-09752-4.

## Background

Gestational diabetes mellitus (GDM) is defined as hyperglycemia that is first detected during pregnancy and currently represents the most common medical complication in pregnancy [1]. In recent years, Due to the rising proportion of advanced maternal age and growing obesity rates, the prevalence of GDM has continued to increase [2]. According to the International Diabetes Federation (IDF), the prevalence of GDM in China reached 15.7% in 2024 [3]. GDM exerts both short-term and long-term adverse effects on mothers and infants, which also imposing a substantial economic burden for both families and healthcare systems [4].

Currently, many national and international guidelines recommend insulin as the first-line medication during pregnancy, as it does not cross the placental barrier and demonstrates a clear glucose-lowering effect [2]. However, insulin therapy still faces several challenges in real-world practice, including increased treatment costs and higher risk of hypoglycemia [5, 6]. Furthermore, some patients are unable to use insulin safely and correctly because of physical constraints or limited understanding [7].

Therefore, non-insulin medications such as metformin, are increasingly used during pregnancy, as they are easier to take and are often associated with better adherence [8]. In some countries, their use has even exceeded that of insulin [9, 10]. However, the effectiveness and safety of metformin during pregnancy remain subjects of debate. While some studies suggest that metformin may provide better glycemic control than insulin, others report no significant differences, and findings regarding adverse pregnancy outcomes remain inconsistent [9, 11, 12].

In addition, most of the existing evidence is derived from randomized controlled trials (RCTs) [13, 14]. Although RCTs maintain high internal validity in terms of causal inference, they still have certain limitations compared with retrospective cohort studies. Firstly, RCTs usually employ strict inclusion and exclusion criteria, resulting in highly selected study populations. This may lead to discrepancies between trial participants and patients encountered in real-world clinical practice, thereby limiting the external generalizability of the findings. Secondly, RCTs often have relatively limited sample sizes and shorter follow-up durations, which may reduce their ability to adequately assess rare adverse pregnancy outcomes. Thirdly, RCTs typically report intention-to-treat (ITT) effects and may not fully account for changes in exposure status, such as the addition of or switch to other glucose-lowering medications among participants initially randomized to metformin treatment due to inadequate glycemic control. This can obscure the accurate evaluation of the real-world effectiveness of specific treatment strategies.

Therefore, this study aimed to evaluate glycemic control and the risk of adverse pregnancy outcomes between the insulin initiation group and the metformin initiation group among women with GDM. The findings are expected to provide evidence-based support for optimizing glucose-lowering treatment strategies in women with GDM and to inform clinical management pathways during pregnancy with real-world evidence.

## Methods

### Data source

This retrospective observational study utilized electronic medical records from a provincial healthcare database in Eastern China. The database integrates multidimensional longitudinal information, including anonymized demographic characteristics, outpatient and inpatient diagnostic records, prescription records, laboratory test results, imaging examinations, and other healthcare service-related data.

### Study population

Women diagnosed with GDM between January 1, 2018 and October 31, 2025 were included in this study. GDM was identified using International Classification of Diseases, 10th Revision (ICD-10) codes (O24.4 or O24.9) and/or text diagnosis records [15]. Women with a diagnosis of diabetes before pregnancy (E10-E14) [16] or with prior use of glucose-lowering medications were excluded (the ATC codes of major glucose-lowering medications are presented in Additional file 1: Table S1).

Eligible patients met the following criteria. Firstly, the total recorded duration of pregnancy in the database had to be at least 20 weeks. Secondly, there had to be at least one prescription record for a glucose-lowering medication between the last menstrual period (LMP) and the end of pregnancy. Thirdly, the age had to be between 18 and 50 years. The exclusion criteria were as follows. Conversely, patients were excluded if gestational age data were missing or pregnancy dates were clearly incorrect. In addition, patients were excluded if they had initiated glucose-lowering medications other than insulin or metformin, had multiple pregnancies, or had incomplete glucose-related test records.

The index date was defined the as date of the first prescription for a glucose-lowering medications during pregnancy. The follow-up period commenced on the index date and continued until the end of pregnancy, loss to follow-up, death, or the database cutoff date (October 31, 2025), whichever occurred first.

Complete blood glucose-related records required both the glycated hemoglobin (HbA1c) value at enrollment and at the end of pregnancy. The extraction rules were as follows. The HbA1c at enrollment had to have a measurement time between the date of the LMP and the index date. From records that met this condition, we selected the test result closest to the index date, and the measurement time had to be within 90 days before the index date. The HbA1c at the end of pregnancy had to have a measurement time between the index date and the end of pregnancy. From records that met this condition, we selected the test result closest to the end of pregnancy date, and the measurement time had to be within 90 days before the end of pregnancy date. The participant selection process is summarized in the screening flow chart (Additional file 1: Figure S1).

### Exposure and outcomes

This study used the intention-to-treat (ITT) principle to define exposure. According to the type of the first prescription of glucose-lowering medications during pregnancy, participants were divided into two independent and mutually exclusive exposure groups: the insulin-initiation group (control group) and the metformin-initiation group. Grouping was based only on the initial treatment at the index date. Changes in treatment after the index date did not affect the original group assignment.

The outcomes included glycemic control and adverse pregnancy outcomes. Glycemic control was evaluated by the HbA1c level at the end of pregnancy, the change in HbA1c from baseline to the end of pregnancy, and the proportion of patients reaching the target at the end of pregnancy. For patients with gestational diabetes mellitus, the HbA1c control target during pregnancy was set at 6.0% [17, 18]. Adverse pregnancy outcomes included cesarean delivery, preterm birth (live birth before 37 weeks of pregnancy), postpartum hemorrhage (PPH), premature rupture of membranes (PROM), preeclampsia, polyhydramnios, oligohydramnios, umbilical cord around the neck, and macrosomia. All adverse pregnancy outcomes were determined based on ICD-10 diagnostic codes and/or text diagnostic information (Additional file 1: Table S2). The observation period for each outcome ended at the following times: for preterm birth, at 37 weeks of pregnancy; for other outcomes, at the end of pregnancy.

### Covariates

This study included information on age, gestational weeks, baseline HbA1c, number of dependent children, marriage, employment status, and types of medical insurance. Age was determined based on patients’ identification information, while the remaining variables were self-reported by patients. Age in this study was categorized into 18–24 years, 25–29 years, 30–34 years, 35–39 years, and 40 years and above. The baseline HbA1c was defined as the glycemic status at the time of the first prescription of glucose-lowering medication during pregnancy. The number of dependent children was categorized as 0 or 1 and above. Marriage status was categorized as married and others. Employment status was categorized as employed, unemployed, and others. Medical insurance was categorized as urban employee basic medical insurance (UEBMI), urban and rural resident basic medical insurance (URRBMI), and Out-of-pocket payment (OOP).

In addition, endocrine comorbidities were included as covariates, including history of infertility treatment, hypertension, dyslipidemia, hypothyroidism, polycystic ovarian syndrome, and obesity. These comorbidities were identified based on ICD-10 diagnostic codes and/or text diagnosis records (Additional file 1: Table S3).

### Statistical analysis

Baseline characteristics were described descriptively across treatment cohorts. Continuous variables were expressed as mean ± standard deviation (mean ± SD), and categorical variables were presented as counts (percentages). To evaluate the effects of the insulin initiation group and the metformin group on glycemic control and adverse pregnancy outcomes, and to reduce selection bias caused by differences in baseline characteristics (such as age, baseline HbA1c, gestational weeks, and comorbidities), this study applied the Overlap Weighting (OW) method for propensity score adjustment. Missing values were handled using Multiple Imputation by Chained Equations (MICE). Propensity scores were estimated using multivariable logistic regression, and weights of 1–PS were assigned to the metformin group and PS to the insulin group to optimize the representativeness of individuals in the overlapping region. Covariates included in the model were age, baseline HbA1c, gestational weeks, number of dependent children, marriage status, employment status, medical insurance type, and endocrine comorbidities. The Standardized Mean Difference (SMD) was used to assess covariate balance between groups, with SMD < 0.1 considered indicative of good balance.

For continuous glycemic outcomes (HbA1c at the end of pregnancy and change in HbA1c), weighted linear regression models were used to analyze differences between the metformin group and the insulin group. For binary outcomes (HbA1c achievement rate at the last measurement during pregnancy), weighted logistic regression models were used to calculate odds ratios (OR). Gestational weeks at enrollment and gestational weeks at the time of HbA1c measurement at the end of pregnancy were included as covariates in the model to indirectly anchor the gestational time axis. To evaluate the risk of adverse pregnancy outcomes in the metformin group compared with the insulin group, time-to-event analysis based on gestational week time scale was applied. Left-truncation was used, meaning observation started from the gestational week at treatment initiation and ended at the gestational week when the outcome occurred or pregnancy ended (whichever came first). Person-weeks of follow-up (total follow-up weeks for the entire group), mean follow-up weeks (average follow-up weeks per person in the group. Incidence), and incidence (per 100 person-weeks) were reported. Time-to-event outcomes were analyzed using a weighted Cox proportional hazards model. The hazard ratio (HR) and its 95% confidence interval (CI) were reported after adjustment for baseline characteristics. The proportional hazards (PH) assumption of the Cox model was tested using the Schoenfeld residual test. Analyses of glycemic outcomes and adverse pregnancy outcomes strictly followed the ITT principle. All time-based analyses used gestational weeks as the time axis.

Subgroup analyses were performed according to baseline HbA1c level, categorized as < 6.0% and ≥ 6.0% based on the recommended glycemic target for women with GDM [17, 18], and gestational age at treatment initiation, categorized as the first trimester (< 14 gestational weeks), second trimester (14–27 + 6 gestational weeks), and third trimester (≥ 28 gestational weeks) [19]. Overlap weighting was applied within each subgroup for the corresponding analyses. Effect modification was assessed by including interaction terms between treatment group and subgroup variables in weighted regression models, and corresponding P values for interaction were reported.

### Sensitivity analysis

To test the robustness of our findings, we conducted a per-protocol (PP) analysis that was restricted to women who continued the initially initiated treatment strategy throughout follow-up, without treatment escalation to or switching across the alternative class. Specifically, women in the metformin initiation group were required to remain on metformin therapy with no subsequent initiation of other glucose-lowering medications, while women in the insulin initiation group were required to remain on insulin therapy with no subsequent initiation of other glucose-lowering medications. We then repeated the primary analyses to evaluate glycemic control and adverse pregnancy outcomes in this PP cohort. For glycemic outcomes, individuals who changed treatment before blood glucose measurement were excluded, and overlap weighting was reapplied to maintain balance between groups. For adverse pregnancy outcomes, time-point censoring was used. Patients who changed treatment were censored on the day of treatment change, and follow-up time was counted from treatment initiation to the date of change. The original weighting coefficients were used in the Cox regression. All statistical analyses were performed using Stata 17.0 and R 4.4.2.

### Ethics

The study protocol was reviewed and approved by the Institutional Review Board of Nanjing Medical University (Approval Number: 20250141). Given that the study utilized anonymized electronic hospital records and did not involve any direct patient contact, the requirement for informed consent was not required.

## Results

### Characteristics of the study population

A total of 7,397 patients with GDM were included in this study (Additional file 1: Figure S1). Among them, 6,922 patients (93.6%) were in the insulin initiation group, and 475 patients (6.4%) were in the metformin initiation group. Before weighting, most baseline covariates showed significant differences between the two groups (SMD > 0.1, Table 1). Compared with the metformin initiation group, the insulin initiation group had a lower gestational week at enrollment (26.0 weeks vs. 28.4 weeks, SMD = 0.27) and a higher HbA1c level (6.4% vs. 5.6%, SMD = 0.64). The proportion of multiparous women (number of dependent children ≥ 1) was higher in the insulin initiation group (23.8% vs. 8.0%, SMD = 0.44), and the proportion of married patients was also higher (72.9% vs. 44.2%, SMD = 0.61). In the metformin initiation group, the proportion of patients with UEBMI was higher (77.3% vs. 64.8%, SMD = 0.28), while the proportion of OOP patients was lower (16.8% vs. 22.3%, SMD = 0.14). For endocrine comorbidities, a history of infertility treatment was more common in the metformin initiation group (19.6% vs. 12.5%, SMD = 0.19), while dyslipidemia was less common (2.3% vs. 5.1%, SMD = 0.15) compared with the insulin initiation group. After overlap weighting, all baseline covariates achieved good balance between the two groups (SMD < 0.1, Table 1).

**Table 1:** Characteristics of the study population

Characteristics	Unweighted			Weighted		
	Insulin (*n* = 6922)	Metformin (*n* = 475)	SMD	Insulin (*n* = 3354)	Metformin (*n* = 468)	SMD
Age, years	32.1 (4.8)	32.4 (4.7)	0.05	32.3 (4.7)	32.3 (4.7)	< 0.10
Gestational age, weeks	26.0 (8.9)	28.4 (9.0)	0.27	28.1 (7.3)	28.1 (9.2)	< 0.10
HbA1c, %	6.4 (1.5)	5.6 (0.8)	0.64	5.7 (0.8)	5.7 (0.8)	< 0.10
Number of dependent children, n (%)						
0	5275 (76.2)	437 (92.0)	0.44	3057 (91.1)	427 (91.1)	< 0.10
≥ 1	1647 (23.8)	38 (8.0)	0.44	297 (8.9)	41 (8.9)	< 0.10
Marriage, n (%)						
Married	5048 (72.9)	210 (44.2)	0.61	1628 (48.5)	227 (48.5)	< 0.10
Others	1874 (27.1)	265 (55.8)	0.61	1726 (51.5)	241 (51.5)	< 0.10
Employment, n (%)						
Employed	3359 (48.5)	187 (39.4)	0.18	1379 (41.1)	192 (41.1)	< 0.10
Unemployed	756 (10.9)	28 (5.9)	0.18	215 (6.4)	30 (6.4)	< 0.10
Others	2807 (40.6)	260 (54.7)	0.29	1760 (52.5)	246 (52.5)	< 0.10
Medical insurance, n (%)						
UEBMI	4483 (64.8)	367 (77.3)	0.28	2537 (75.6)	354 (75.6)	< 0.10
URRBMI	896 (12.9)	28 (5.9)	0.24	219 (6.5)	31 (6.5)	< 0.10
OOP	1543 (22.3)	80 (16.8)	0.14	598 (17.8)	83 (17.8)	< 0.10
Infertility treatment, n (%)	865 (12.5)	93 (19.6)	0.19	641 (19.1)	89 (19.1)	< 0.10
Hypertension, n (%)	600 (8.7)	50 (10.5)	0.06	353 (10.5)	49 (10.5)	< 0.10
Dyslipidemia, n (%)	354 (5.1)	11 (2.3)	0.15	82 (2.5)	11 (2.5)	< 0.10
Hypothyroidism, n (%)	581 (8.4)	53 (11.2)	0.09	365 (10.9)	51 (10.9)	< 0.10
Polycystic ovarian syndrome, n (%)	170 (2.5)	15 (3.2)	0.04	103 (3.1)	14 (3.1)	< 0.10
Obesity, n (%)	607 (8.8)	43 (9.1)	0.01	307 (9.2)	43 (9.2)	< 0.10

*Abbreviations*: *UEBMI* Urban employee basic medical insurance, *URRBMI* Urban and rural resident basic medical insurance, *OOP* Out-of-pocket payment

### Comparison of glycemic control

In the unweighted analysis, the HbA1c level at the end of pregnancy was slightly lower in the metformin initiation group than in the insulin initiation group (β: -0.30, 95% CI: -0.38 to -0.22; *P* < 0.001, Table 2). However, initiation of metformin was associated with a smaller reduction in HbA1c than insulin (β: 0.47, 95% CI: 0.36 to 0.58; *P* < 0.001). In addition, the target achievement rate of HbA1c (< 6.0%) was significantly higher in the metformin initiation group (OR: 1.93, 95% CI: 1.57–2.37; *P* < 0.001, Table 3). After adjustment for baseline differences, no statistically significant differences were observed between the two groups in HbA1c level at the end of pregnancy, the change in HbA1c, and target achievement (Tables 2 and 3).

**Table 2:** Comparison of glycemic levels between groups

	Unadjusted			Adjusted		
	Insulin	Metformin	Coefficient (95% CI)	Insulin	Metformin	Coefficient (95% CI)
HbA1c at the end of pregnancy (%)	6.00 ± 0.88	5.70 ± 0.59	-0.30^***^(-0.38, -0.22)	5.71 ± 0.65	5.72 ± 0.60	0.01 (-0.05, 0.06)
Change in HbA1c (%)	-0.41 ± 1.25	0.06 ± 0.75	0.47^***^(0.36, 0.58)	0.01 ± 0.65	0.03 ± 0.77	0.01 (-0.05, 0.06)

*Abbreviations*: *CI* Confidence interval

*p<0.05, **p<0.01, ***p<0.001

**Table 3:** Comparison of glycemic control rate (< 6.0%) between groups

	Unadjusted	OR (95% CI)	Adjusted	OR (95% CI)
Insulin	56.4%	1.93^***^(1.57, 2.37)	69.7%	1.07 (0.83, 1.38)
Metformin	71.4%		69.8%	

*Abbreviations*: *OR* Odds ratios, *CI* Confidence interval

*p<0.05, **p<0.01, ***p<0.001

### Risk of adverse pregnancy outcomes

In the unweighted analysis, women initiating metformin therapy had a significantly lower risk of cesarean section (HR: 0.70, 95% CI: 0.57–0.86; *P* < 0.001), PROM (HR: 0.62, 95% CI: 0.45–0.86; *P* = 0.005), and macrosomia (HR: 0.52, 95% CI: 0.35–0.79; *P* = 0.002) compared with those initiating insulin (Additional file 1: Table S4).

After weighting, follow-up time was comparable between groups. The mean follow-up duration was 11.4 weeks per person for cesarean section (Table 4). The crude incidence of cesarean section was 1.85 per 100 person-weeks (95% CI: 1.46–2.24) in the metformin initiation group and 2.39 per 100 person-weeks (95% CI: 1.94–2.84) in the insulin initiation group. For PROM, the mean follow-up time was 11.6 weeks per person, with crude incidence rates of 0.72 per 100 person-weeks (95% CI: 0.48–0.96) and 1.07 per 100 person-weeks (95% CI: 0.77–1.37) in the metformin and insulin initiation groups, respectively. For macrosomia, the mean follow-up time was 11.7 weeks per person, and the crude incidence was 0.44 per 100 person-weeks (95% CI: 0.25–0.63) in the metformin initiation group versus 0.68 per 100 person-weeks (95% CI: 0.44–0.91) in the insulin initiation group.

**Table 4:** Comparison of weighted risk of adverse pregnancy outcomes between groups

	No. of events	Person-weeks of follow-up	Mean follow-up weeks	Incidence (95% CI)	HR (95% CI)
Cesarean delivery					
Insulin	109	4570	11.42	2.39 (1.94–2.84)	Reference
Metformin	86	4654	11.40	1.85 (1.46–2.24)	0.77^*^(0.62–0.95)
Preterm birth					
Insulin	48	3466	9.27	1.38 (0.99–1.77)	Reference
Metformin	40	3500	8.97	1.14 (0.79–1.50)	0.86 (0.63–1.18)
PPH					
Insulin	14	4772	11.76	0.30 (0.14–0.45)	Reference
Metformin	9	4806	11.78	0.20 (0.07–0.32)	0.62 (0.33–1.16)
PROM					
Insulin	50	4674	11.64	1.07 (0.77–1.37)	Reference
Metformin	34	4741	11.62	0.72 (0.48–0.96)	0.66^*^(0.48–0.93)
Preeclampsia					
Insulin	30	4664	11.56	0.65 (0.42–0.88)	Reference
Metformin	38	4651	11.49	0.81 (0.55–1.07)	1.23 (0.89–1.71)
Polyhydramnios					
Insulin	7	4771	11.77	0.15 (0.04–0.26)	Reference
Metformin	5	4803	11.77	0.10 (0.01–0.18)	0.73 (0.27–1.98)
Oligohydramnios					
Insulin	15	4747	11.78	0.32 (0.16–0.48)	Reference
Metformin	11	4807	11.78	0.23 (0.10–0.37)	0.71 (0.40–1.26)
Umbilical cord around the neck					
Insulin	60	4641	11.61	1.29 (0.96–1.61)	Reference
Metformin	53	4725	11.58	1.13 (0.82–1.43)	0.87 (0.67–1.14)
Macrosomia					
Insulin	32	4756	11.72	0.68 (0.44–0.91)	Reference
Metformin	21	4798	11.76	0.44 (0.25–0.63)	0.61^*^(0.40–0.93)

No. of events: number of events. Person-weeks of follow-up: total follow-up weeks for the entire group. Mean follow-up weeks: average follow-up weeks per person in the group. Incidence: per 100 person-week

*Abbreviations*: *PPH* Postpartum hemorrhage, *PROM* Premature rupture of membranes, *CI* Confidence interval, *HR* Hazard ratio

^*^p < 0.05, ^**^p < 0.01, ^***^p < 0.001

In weighted Cox proportional hazards models using gestational weeks as the time scale, initiation of metformin therapy remained associated with a lower risk of cesarean section (HR: 0.77, 95% CI: 0.62–0.95; *P* = 0.017), PROM (HR: 0.66, 95% CI: 0.48–0.93; *P* = 0.016), and macrosomia (HR: 0.61, 95% CI: 0.40–0.93; *P* = 0.021) compared with insulin initiation. No statistically significant differences were observed for other adverse pregnancy outcomes.

### Subgroup analyses

#### Subgroup analyses according to baseline HbA1c

Significant interactions were observed between treatment strategy and baseline HbA1c category (all P for interaction < 0.01, Additional file 1: Table S5). For HbA1c at the end of pregnancy, among women with baseline HbA1c < 6.0%, metformin initiation was associated with slightly higher HbA1c compared with insulin initiation (β: 0.05, 95% CI: 0.01–0.10; *P* = 0.043). In contrast, among women with baseline HbA1c ≥ 6.0%, initiation of metformin was associated with lower HbA1c than insulin (β: -0.15, 95% CI: -0.27 to -0.03; *P* = 0.014). Similar patterns of effect modification were observed for change in HbA1c and glycemic control rate (< 6.0%) (all P for interaction < 0.01).

For adverse pregnancy outcomes, no statistically significant interactions were observed between treatment strategy and baseline HbA1c category (all P for interaction > 0.05, Additional file 1: Table S6).

#### Subgroup analyses according to gestational age at treatment initiation

Subgroup analyses by gestational age at treatment initiation showed no significant interactions between treatment strategy and gestational age for HbA1c at the end of pregnancy, change in HbA1c, or glycemic control rate (< 6.0%) (all P for interaction > 0.05, Additional file 1: Table S5).

Similarly, for adverse pregnancy outcomes, no significant interactions were observed between treatment strategy and gestational age subgroup for any outcome (all P for interaction > 0.05, Additional file 1: Table S7).

### Sensitivity analyses

During follow-up, treatment changes were observed in both groups. In the metformin initiation group, 74 women (15.6%) subsequently initiated additional glucose-lowering medications. In the insulin initiation group, 229 women (3.3%) subsequently initiated non-insulin glucose-lowering medications (i.e., added or switched to other glucose-lowering medications).

To assess the impact of these treatment changes, we performed a per-protocol (PP) analysis restricted to women who remained on their initially initiated treatment strategy throughout follow-up, without crossover to the alternative class. The effect estimates from the PP analysis were consistent with those from the primary intention-to-treat (ITT) analysis for both glycemic control and adverse pregnancy outcomes, supporting the robustness of our findings (Additional file 1: Tables S8-S10).

## Discussion

This retrospective cohort study compared initial metformin versus insulin therapy among women with GDM in China, focusing on glycemic control and adverse pregnancy outcomes. After balancing baseline characteristics, we observed no evidence of a difference in glycemic control between groups, as reflected by HbA1c at the end of pregnancy, changes in HbA1c, and achievement of the HbA1c target (< 6.0%). In contrast, women initiating metformin therapy experienced lower risks of selected adverse pregnancy outcomes, including cesarean section, PROM, and macrosomia. Subgroup analyses demonstrated significant interactions between treatment strategy and baseline HbA1c category for glycemic outcomes (all P for interaction < 0.01). Findings were consistent across sensitivity analyses, supporting the robustness of the primary results. Overall, initiation of metformin was associated with glycemic control comparable to that of insulin.

### Comparison with previous studies

Compared with previous studies, the conclusions of this study are consistent to some extent. Regarding glycemic control, several previous RCTs have shown similar effectiveness between the insulin treatment group and the metformin treatment group in improving glycemic levels [12, 20]. After adjustment using propensity score overlap weighting, this study also found no significant difference in glycemic control between the two groups, further supporting existing evidence that metformin provides glycemic control comparable to insulin. Subgroup analyses suggested that baseline HbA1c may influence the relative effectiveness of different treatment strategies, although the overall effect was modest. Among women with HbA1c < 6.0%, insulin was associated with slightly better glycemic control, whereas among those with HbA1c ≥ 6.0%, metformin showed a numerical trend toward lower glucose levels. This finding is biologically plausible, as higher HbA1c generally reflects a more pronounced insulin-resistant state [21–23], which may respond more favorably to metformin through suppression of hepatic gluconeogenesis and improvement in peripheral insulin sensitivity [24].

For pregnancy outcomes, we found that initiation of metformin therapy was associated with a lower risk of cesarean section, in line with several high-quality evidence syntheses and comparative studies [11, 25]. However, this finding may have been partly influenced by differences in delivery preferences. Based on baseline characteristics, we found that the proportion of primiparous women (number of dependent children = 0) was higher in the metformin initiation group. Previous studies have reported that multiparous women are more likely to choose cesarean section than primiparous women, while the preference for elective cesarean section is lower among primiparous women [26, 27]. Although this study adjusted for parity in the weighted model, residual confounding from unobserved factors such as delivery preference cannot be completely excluded. We also observed a lower risk of macrosomia among women initiating metformin therapy, consistent with a UK cohort study [28]. Evidence from multicenter studies has suggested that metformin can cross the placenta and may improve maternal–fetal glucose metabolism and insulin resistance, which could contribute to a reduced risk of macrosomia [29]. In addition, the risk of PROM was lower among women initiating metformin therapy. Evidence for this outcome remains limited because PROM has not been a primary endpoint in most RCTs and is relatively uncommon, reducing power to detect differences. Therefore, our large real-world cohort adds epidemiological evidence for this association. From a mechanistic standpoint, PROM is linked to infection and inflammatory pathways [30], and metformin has been shown to have anti-inflammatory effects [31], which may help maintain membrane integrity. However, further clinical studies are needed to validate this association and provide evidence for clinical decision-making.

### Implications of the study

In line with current guideline recommendations, international guidelines including those of American Diabetes Association (ADA) and American College of Obstetricians and Gynecologists (ACOG) continue to recommend insulin as the first-line pharmacological treatment for GDM, mainly because it does not cross the placenta and has relatively robust long-term safety evidence [32, 33]. However, some countries have begun to accept non-insulin medications such as metformin as alternative options in specific situations, particularly for patients who refuse or cannot use insulin appropriately [34]. Our findings provide additional evidence that may help inform treatment strategies for GDM.

It is noteworthy that 15.6% of patients in the metformin initiation group added or switched to other glucose-lowering medications during follow-up, suggesting that some patients may still require treatment intensification when disease progression or insufficient glycemic control occurs. Therefore, when metformin therapy is chosen as the initial strategy, clinicians should strengthen dynamic blood glucose monitoring and adjust treatment promptly when necessary to ensure maternal and neonatal safety and achieve optimal pregnancy outcomes.

### Strengths and limitations

This study has several strengths. Firstly, it leveraged a large electronic medical record database from China, enhancing the generalizability of the findings and real-world representativeness. Secondly, we used propensity score overlap weighting and assessed covariate balance with standardized mean differences, improving comparability between treatment groups. Thirdly, time-to-event analysis used gestational weeks as the time scale and applied left-truncation to handle differences in treatment initiation timing, better reflecting the temporal characteristics of pregnancy and improving the interpretation of treatment timing and gestational effects on outcomes. Finally, results were consistent across multiple sensitivity analyses, including per-protocol and restricted-exposure definitions, supporting the robustness of the primary findings.

Several limitations should be considered. Despite extensive adjustment for potential confounders, residual confounding due to unmeasured or unavailable variables cannot be excluded. Several clinically relevant maternal factors may significantly affect pregnancy outcomes, including pre-pregnancy body mass index (BMI), gestational weight gain, dietary habits, and physical activity [35–37]. In addition, fetal growth percentiles have been consistently associated with perinatal outcomes [38, 39]. However, due to limitation of the database, we were not able to account for these characteristics in the analyses. Accordingly, our findings should be interpreted with caution, and future studies incorporating more comprehensive maternal, fetal, and pregnancy-related data are warranted.

## Conclusion

In this large-scale, real-world cohort of Chinese women with GDM, the metformin initiation group achieved glycemic control comparable to the insulin initiation group after rigorous adjustment for baseline confounders using propensity score overlap weighting. Furthermore, initiation of metformin was associated with lower risks of cesarean delivery, PROM, and macrosomia. While these results advocate for more individualized management in GDM, further high-quality prospective studies and long-term follow-up are warranted to evaluate the short- and long-term effects of these treatment strategies on maternal and neonatal health outcomes.

## Supplementary Information

Additional file 1: Figure S1. Flowchart of participants in the study cohorts. Table S1. The ATC codes of major glucose-lowering medications. Table S2. ICD-10 codes related to adverse pregnancy outcomes. Table S3. ICD-10 codes related to endocrine comorbidities. Table S4. Comparison of unweighted risk of adverse pregnancy outcomes between groups. Table S5. Overlap-weighted subgroup analyses of glycemic outcomes stratified by baseline HbA1c and gestational age. Table S6. Overlap-weighted subgroup analyses of adverse pregnancy outcomes stratified by baseline HbA1c. Table S7. Overlap-weighted subgroup analyses of adverse pregnancy outcomes stratified by gestational age at treatment initiation. Table S8. Results of per-protocol linear regression comparing glycemic levels between groups. Table S9. Results of per-protocol logistic regression comparing glycemic control rate (<6.0%) between groups. Table S10. Results of per-protocol Cox regression comparing adverse pregnancy outcomes between groups.

## Data Availability

The individual-level data analysed in this study are not publicly available because they contain sensitive participant information and are subject to restrictions arising from the study ethics approval and applicable data-protection requirements. The corresponding author (DY) serves as the designated data custodian for this study. The statistical code used in this study is available from the corresponding author upon reasonable request.

## Abbreviations

GDM: Gestational diabetes mellitus
HbA1c: Glycated hemoglobin
IDF: International Diabetes Federation
RCTs: Randomized controlled trials
ITT: Intention-to-treat
LMP: Last menstrual period
PPH: Postpartum hemorrhage
PROM: Premature rupture of membranes
UEBMI: Urban employee basic medical insurance
URRBMI: Urban and rural resident basic medical insurance
OOP: Out-of-pocket payment
OW: Overlap Weighting
MICE: Multiple Imputation by Chained Equations
SMD: Standardized Mean Difference
OR: Odds ratios
HR: Hazard ratio
CI: Confidence interval
PH: Proportional hazards
PP: Per-protocol
ADA: American Diabetes Association
ACOG: American College of Obstetricians and Gynecologists
EFW: Estimated fetal weight
