# Supplementary material for: Comparative effectiveness of initiating insulin versus metformin therapy for gestational diabetes mellitus in China: a retrospective cohort study
Source: BMC Pregnancy Childbirth. 2026 Jul 31;26:1228. doi: 10.1186/s12884-026-09752-4 (PMC13711749; doi:10.1186/s12884-026-09752-4)
Supplement: Supplementary file 1 [file 12884_2026_9752_MOESM1_ESM.docx]

# Supplementary Information

Additional file 1: Figure S1and Table S1-S10

Figure S1. Flowchart of participants in the study cohorts.

Table S1. The ATC codes of major glucose-lowering medications

Table S2. ICD-10 codes related to adverse pregnancy outcomes.

Table S3. ICD-10 codes related to endocrine comorbidities.

Table S4. Comparison of unweighted risk of adverse pregnancy outcomes between groups.

Table S5. Overlap-weighted subgroup analyses of glycemic outcomes stratified by baseline HbA1c and gestational age.

Table S6. Overlap-weighted subgroup analyses of adverse pregnancy outcomes stratified by baseline HbA1c.

Table S7. Overlap-weighted subgroup analyses of adverse pregnancy outcomes stratified by gestational age at treatment initiation.

Table S8. Results of per-protocol linear regression comparing glycemic levels between groups.

Table S9. Results of per-protocol logistic regression comparing glycemic control rate (<6.0%) between groups.

Table S10. Results of per-protocol Cox regression comparing adverse pregnancy outcomes between groups.

## Figure S1. Flowchart of participants in the study cohorts.

## Table S1. The ATC codes of major glucose-lowering medications.

| **Agents** | **ATC** **codes** |
| --- | --- |
| Insulins and analogues | A10A |
| Biguanides | A10BA* |
| Sulfonylureas | A10BB |
| Alpha glucosidase inhibitors | A10BF |
| Thiazolidinediones | A10BG |
| Glucagon-like peptide-1 (GLP-1) analogues | A10BJ |
| Sodium-glucose co-transporter 2 (SGLT2) inhibitors | A10BK |
| Dipeptidyl peptidase 4 (DPP-4) inhibitors | A10BH |
| Combinations of oral blood glucose lowering medications | A10BD |
| Other blood glucose lowering drugs (excluded insulins) | A10BX |

**Note:** *All records classified under the ATC code A10BA (biguanides) were metformin (A10BA02).

## Table S2. ICD-10 codes related to adverse pregnancy outcomes.

| **Outcomes** | **ICD-10 code** |
| --- | --- |
| Cesarean delivery | O82.0、O82.1、O82.2、O82.8、O82.9 |
| Preterm birth | O60.0、O60.1、O60.2、O60.3 |
| PPH | O72.1、O72.2 |
| PROM | O42.0、O42.1、O42.2、O42.9 |
| Preeclampsia | O13.x02、O14.0、O14.1、O14.9、O15.0、O15.1、O15.2、O15.9 |
| Polyhydramnios | O40.x00x001 |
| Oligohydramnios | O41.000 |
| Umbilical cord around the neck | O69.1、P02.5 |
| Macrosomia | O36.601、O33.501、O66.201、P08.000 |

**Abbreviations:** PPH, postpartum hemorrhage; PROM, premature rupture of membranes.

## Table S3. ICD-10 codes related to endocrine comorbidities.

| **Endocrine comorbidities** | **ICD-10 code** |
| --- | --- |
| Infertility treatment | Z31.1、Z31.2、Z31.3、Z31.8、Z31.9 |
| Hypertension | I10、I11、I12、I13、I14、I15 |
| Dyslipidemia | E78 |
| Hypothyroidism | E01.8、E03、E89.0、O99.215 |
| Polycystic ovarian syndrome | E28.2 |
| Obesity | E66 |

## Table S4. Comparison of unweighted risk of adverse pregnancy outcomes between groups.

|  | No. of events | Person-weeks of follow-up | Mean follow-up weeks | | Incidence (95% CI) | HR (95% CI) |
| --- | --- | --- | --- | --- | --- | --- |
| Cesarean delivery |  |  | |  |  |  |
| Insulin | 2013 | 91326 | | 13.48 | 2.20 (2.11-2.30) | Reference |
| Metformin | 98 | 5314 | | 11.19 | 1.84 (1.48-2.21) | 0.70^***^(0.57-0.86) |
| Preterm birth |  |  | |  |  |  |
| Insulin | 889 | 73595 | | 11.53 | 1.21 (1.13-1.29) | Reference |
| Metformin | 46 | 3970 | | 8.76 | 1.16 (0.82-1.49) | 0.82 (0.61-1.11) |
| PPH |  |  | |  |  |  |
| Insulin | 233 | 95581 | | 13.90 | 0.24 (0.21-0.28) | Reference |
| Metformin | 11 | 5490 | | 11.56 | 0.20 (0.08-0.32) | 0.65 (0.35-1.21) |
| PROM |  |  | |  |  |  |
| Insulin | 937 | 93414 | | 13.70 | 1.00 (0.94-1.07) | Reference |
| Metformin | 38 | 5416 | | 11.40 | 0.70 (0.48-0.92) | 0.62^**^(0.45-0.86) |
| Preeclampsia |  |  | |  |  |  |
| Insulin | 694 | 92965 | | 13.59 | 0.75 (0.69-0.80) | Reference |
| Metformin | 42 | 5314 | | 11.28 | 0.79 (0.55-1.03) | 1.15 (0.84-1.59) |
| Polyhydramnios |  |  | |  |  |  |
| Insulin | 173 | 95289 | | 13.88 | 0.18 (0.15-0.21) | Reference |
| Metformin | 5 | 5485 | | 11.55 | 0.09 (0.01-0.17) | 0.60 (0.24-1.48) |
| Oligohydramnios |  |  | |  |  |  |
| Insulin | 250 | 95096 | | 13.90 | 0.26 (0.23-0.30) | Reference |
| Metformin | 13 | 5490 | | 11.56 | 0.24 (0.11-0.37) | 0.77 (0.44-1.36) |
| Umbilical cord around the neck |  |  | |  |  |  |
| Insulin | 1026 | 93198 | | 13.72 | 1.10 (1.03-1.17) | Reference |
| Metformin | 60 | 5394 | | 11.36 | 1.11 (0.83-1.39) | 0.82 (0.62-1.07) |
| Macrosomia |  |  | |  |  |  |
| Insulin | 735 | 94838 | | 13.82 | 0.78 (0.72-0.83) | Reference |
| Metformin | 24 | 5481 | | 11.54 | 0.44 (0.26-0.61) | 0.52^**^(0.35-0.79) |

**Note:** ^*^p<0.05, ^**^p<0.01, ^***^p<0.001. No. of events: number of events. Person-weeks of follow-up: total follow-up weeks for the entire group. Mean follow-up weeks: average follow-up weeks per person in the group. Incidence: per 100 person-week.

**Abbreviations:** PPH, postpartum hemorrhage; PROM, premature rupture of membranes; CI, Confidence interval; HR, Hazard ratio.

## Table S5. Overlap-weighted subgroup analyses of glycemic outcomes stratified by baseline HbA1c and gestational age.

| HbA1c at the end of pregnancy (%) | | | | | | |  |
| --- | --- | --- | --- | --- | --- | --- | --- |
| Subgroup | Insulin | Metformin | Coefficient (95% CI) | P | P interaction | |  |
| Baseline HbA1c |  |  |  |  | p<0.001 | |  |
| ＜6.0% | 5.50±0.50 | 5.58±0.50 | 0.05 (0.01, 0.10) | p=0.043 |  | |  |
| ≥6.0% | 6.27±0.74 | 6.11±0.65 | -0.15 (-0.27, -0.03) | p=0.014 |  | |  |
| Gestational age |  |  |  |  | p=0.178 | |  |
| First trimester | 5.73±0.74 | 5.61±0.63 | -0.12 (-0.30, 0.06) | p=0.192 |  | |  |
| Second trimester | 5.82±0.70 | 5.82±0.69 | -0.03 (-0.16, 0.10) | p=0.646 |  | |  |
| Third trimester | 5.66±0.62 | 5.71±0.56 | 0.03 (-0.02, 0.08) | p=0.239 |  | |  |
| Change in HbA1c (%) | | | | | | |  |
| Subgroup | Insulin | Metformin | Coefficient (95% CI) | P | P interaction | |  |
| Baseline HbA1c |  |  |  |  | p<0.001 | |  |
| ＜6.0% | 0.18±0.41 | 0.26±0.44 | 0.05 (0.01, 0.10) | p=0.043 |  | |  |
| ≥6.0% | -0.42±0.94 | -0.58±1.07 | -0.15 (-0.27, -0.03) | p=0.014 |  | |  |
| Gestational age |  |  |  |  | p=0.178 | |  |
| First trimester | -0.77±1.12 | -0.90±1.43 | -0.12 (-0.30, 0.06) | p=0.192 |  | |  |
| Second trimester | 0.01±0.70 | 0.01±0.83 | -0.03 (-0.16, 0.10) | p=0. 646 |  | |  |
| Third trimester | 0.11±0.42 | 0.16±0.53 | 0.03 (-0.02, 0.08) | p=0.239 |  | |  |
| Glycemic control rate (<6.0%) | | | | | | | |
| Subgroup | Insulin | Metformin | OR (95% CI) | P | | P interaction | |
| Baseline HbA1c |  |  |  |  | | p=0.009 | |
| ＜6.0% | 84.0% | 79.5% | 0.80 (0.58, 1.12) | p=0.188 | |  | |
| ≥6.0% | 32.1% | 43.5% | 1.79 (1.19, 2.70) | p=0.005 | |  | |
| Gestational age |  |  |  |  | | p=0.148 | |
| First trimester | 69.6% | 80.2% | 2.04 (0.89, 4.68) | p=0.093 | |  | |
| Second trimester | 63.9% | 62.4% | 1.08 (0.63, 1.85) | p=0.775 | |  | |
| Third trimester | 72.1% | 70.1% | 0.92 (0.66, 1.30) | p=0.650 | |  | |

**Abbreviations:** OR, odds ratios; CI, Confidence interval.

## Table S6. Overlap-weighted subgroup analyses of adverse pregnancy outcomes stratified by baseline HbA1c.

| Outcome | Subgroup | HR (95% CI) | P | P interaction |
| --- | --- | --- | --- | --- |
| Cesarean delivery | ＜6.0% | 0.66 (0.70-1.37) | p=0.003 | p=0.082 |
|  | ≥6.0% | 0.98 (0.56-1.60) | p=0.891 |  |
| Preterm birth | ＜6.0% | 0.80 (0.54-1.19) | p=0.273 | p=0.546 |
|  | ≥6.0% | 0.94 (0.56-1.60) | p=0.827 |  |
| PPH | ＜6.0% | 0.51 (0.22-1.18) | p=0.114 | p=0.203 |
|  | ≥6.0% | 0.98 (0.40-2.40) | p=0.973 |  |
| PROM | ＜6.0% | 0.70 (0.48-1.04) | p=0.080 | p=0.955 |
|  | ≥6.0% | 0.68 (0.39-1.19) | p=0.178 |  |
| Preeclampsia | ＜6.0% | 1.28 (0.84-1.97) | p=0.256 | p=0.437 |
|  | ≥6.0% | 1.06 (0.66-1.70) | p=0.821 |  |
| Polyhydramnios | ＜6.0% | 0.44 (0.11-1.72) | p=0.237 | p=0.376 |
|  | ≥6.0% | 1.34 (0.36-4.93) | p=0.662 |  |
| Oligohydramnios | ＜6.0% | 0.70 (0.36-1.37) | p=0.297 | p=0.748 |
|  | ≥6.0% | 0.68 (0.23-2.00) | p=0.482 |  |
| Umbilical cord around the neck | ＜6.0% | 0.82 (0.59-1.12) | p=0.210 | p=0.309 |
|  | ≥6.0% | 0.98 (0.62-1.56) | p=0.930 |  |
| Macrosomia | ＜6.0% | 0.73 (0.43-1.25) | p=0.251 | p=0.472 |
|  | ≥6.0% | 0.60 (0.33-1.10) | p=0.100 |  |

**Abbreviations:** PPH, postpartum hemorrhage; PROM, premature rupture of membranes; CI, Confidence interval; HR, Hazard ratio.

## Table S7. Overlap-weighted subgroup analyses of adverse pregnancy outcomes stratified by gestational age at treatment initiation.

| Outcome | Subgroup | HR (95% CI) | P | P interaction |
| --- | --- | --- | --- | --- |
| Cesarean delivery | First trimester | 0.49 (0.26-0.93) | p=0.028 | p=0.558 |
|  | Second trimester | 0.87 (0.55-1.37) | p=0.551 |  |
|  | Third trimester | 0.78 (0.60-1.02) | p=0.073 |  |
| Preterm birth | First trimester | 1.26 (0.52-3.08) | p=0.612 | p=0.227 |
|  | Second trimester | 1.10 (0.66-1.81) | p=0.721 |  |
|  | Third trimester | 0.70 (0.44-1.11) | p=0.127 |  |
| PPH | First trimester | 0.14 (0.02-0.84) | p=0.032 | p=0.947 |
|  | Second trimester | 0.48 (0.18-1.29) | p=0.145 |  |
|  | Third trimester | 0.64 (0.29-1.45) | p=0.290 |  |
| PROM | First trimester | 0.79 (0.29-2.17) | p=0.651 | p=0.144 |
|  | Second trimester | 0.94 (0.50-1.75) | p=0.834 |  |
|  | Third trimester | 0.55 (0.36-0.85) | p=0. 007 |  |
| Preeclampsia | First trimester | 1.31 (0.53-3.24) | p=0.558 | p=0.404 |
|  | Second trimester | 2.19 (1.32-3.62) | p=0.502 |  |
|  | Third trimester | 0.95 (0.54-1.67) | p=0.864 |  |
| Polyhydramnios | First trimester | 1.47 (0.57-3.79) | p=0.425 | p=0.819 |
|  | Second trimester | 0.47 (0.09-2.39) | p=0.363 |  |
|  | Third trimester | 0.77 (0.21-2.87) | p=0.694 |  |
| Oligohydramnios | First trimester | 0.89 (0.35-2.26) | p=0.814 | p=0.796 |
|  | Second trimester | NA | NA |  |
|  | Third trimester | 0.92 (0.47-1.83) | p=0.815 |  |
| Umbilical cord around the neck | First trimester | 0.92 (0.44-1.95) | p=0.828 | p=0.806 |
|  | Second trimester | 0.46 (0.22-0.95) | p=0.037 |  |
|  | Third trimester | 0.92 (0.68-1.25) | p=0.611 |  |
| Macrosomia | First trimester | 0.13 (0.02-0.76) | p=0.024 | p=0.136 |
|  | Second trimester | 0.62 (0.26-1.49) | p=0.281 |  |
|  | Third trimester | 0.85 (0.51-1.39) | p=0.515 |  |

**Abbreviations:** PPH, postpartum hemorrhage; PROM, premature rupture of membranes; CI, Confidence interval; HR, Hazard ratio.

**Note:** NA indicates that no events were observed in the metformin group for the corresponding subgroup; thus, hazard ratios could not be estimated.

## Table S8. Results of per-protocol linear regression comparing glycemic levels between groups.

|  | Unadjusted | | | Adjusted | | |
| --- | --- | --- | --- | --- | --- | --- |
|  | Insulin | Metformin | Coefficient (95% CI) | Insulin | Metformin | Coefficient (95% CI) |
| HbA1c at the end of pregnancy (%) | 5.99±0.87 | 5.65±0.56 | -0.34^***^(-0.43, -0.26) | 5.66±0.63 | 5.67±0.56 | -0.02 (-0.07, 0.03) |
| Change in HbA1c (%) | -0.39±1.23 | 0.11±0.60 | 0.50^***^(0.38, 0.62) | 0.07±0.55 | 0.08±0.61 | -0.02 (-0.07, 0.03) |

**Note:** ^*^p<0.05, ^**^p<0.01, ^***^p<0.001.

**Abbreviations:** CI, Confidence interval.

## Table S9. Results of per-protocol logistic regression comparing glycemic control rate (<6.0%) between groups.

|  | Unadjusted | OR (95% CI) | Adjusted | OR (95% CI) |
| --- | --- | --- | --- | --- |
| Insulin | 56.8% | 2.17^***^(1.74, 2.74) | 72.2% | 1.13 (0.85, 1.50) |
| Metformin | 74.1% |  | 72.5% |  |

**Note:** ^*^p<0.05, ^**^p<0.01, ^***^p<0.001.

**Abbreviations:** OR, odds ratios; CI, Confidence interval.

## Table S10. Results of per-protocol Cox regression comparing adverse pregnancy outcomes between groups.

|  | No. of events | | Person-weeks of follow-up | | Mean follow-up weeks | | Incidence (95% CI) | | HR (95% CI) | |
| --- | --- | --- | --- | --- | --- | --- | --- | --- | --- | --- |
| Cesarean delivery | |  | |  | |  | |  | |  |
| Insulin | | 107 | | 4478 | | 11.19 | | 2.38 (1.93-2.84) | | Reference |
| Metformin | | 65 | | 3838 | | 9.40 | | 1.71 (1.29-2.12) | | 0.73^*^(0.57-0.94) |
| Preterm birth | |  | |  | |  | |  | |  |
| Insulin | | 47 | | 3406 | | 9.12 | | 1.37 (0.98-1.76) | | Reference |
| Metformin | | 32 | | 2862 | | 7.33 | | 1.12 (0.74-1.51) | | 0.85 (0.60-1.21) |
| PPH | |  | |  | |  | |  | |  |
| Insulin | | 14 | | 4675 | | 11.52 | | 0.30 (0.14-0.46) | | Reference |
| Metformin | | 8 | | 3945 | | 9.67 | | 0.19 (0.06-0.33) | | 0.58 (0.29-1.16) |
| PROM | |  | |  | |  | |  | |  |
| Insulin | | 49 | | 4580 | | 11.41 | | 1.07 (0.77-1.37) | | Reference |
| Metformin | | 32 | | 3876 | | 9.50 | | 0.83 (0.54-1.11) | | 0.80 (0.57-1.13) |
| Preeclampsia | |  | |  | |  | |  | |  |
| Insulin | | 29 | | 4573 | | 11.34 | | 0.64 (0.41-0.87) | | Reference |
| Metformin | | 26 | | 3840 | | 9.49 | | 0.69 (0.43-0.95) | | 1.19 (0.80-1.76) |
| Polyhydramnios | |  | |  | |  | |  | |  |
| Insulin | | 7 | | 4675 | | 11.54 | | 0.15 (0.04-0.26) | | Reference |
| Metformin | | 3 | | 3944 | | 9.66 | | 0.07 (0.00-0.16) | | 0.51 (0.16-1.61) |
| Oligohydramnios | |  | |  | |  | |  | |  |
| Insulin | | 15 | | 4652 | | 11.54 | | 0.32 (0.16-0.48) | | Reference |
| Metformin | | 10 | | 3938 | | 9.65 | | 0.26 (0.10-0.43) | | 0.76 (0.43-1.36) |
| Umbilical cord around the neck | |  | |  | |  | |  | |  |
| Insulin | | 58 | | 4549 | | 11.38 | | 1.27 (0.94-1.60) | | Reference |
| Metformin | | 48 | | 3866 | | 9.47 | | 1.23 (0.88-1.58) | | 0.95 (0.72-1.27) |
| Macrosomia | |  | |  | |  | |  | |  |
| Insulin | | 31 | | 4660 | | 11.49 | | 0.67 (0.44-0.91) | | Reference |
| Metformin | | 18 | | 3931 | | 9.63 | | 0.45 (0.24-0.65) | | 0.63^*^(0.40-0.99) |

**Note:** *p<0.05, **p<0.01, ***p<0.001. No. of events: number of events. Person-weeks of follow-up: total follow-up weeks for the entire group. Mean follow-up weeks: average follow-up weeks per person in the group. Incidence: per 100 person-week.

**Abbreviations:** PPH, postpartum hemorrhage; PROM, premature rupture of membranes; CI, Confidence interval; HR, Hazard ratio.
